# Phenotype Enhancement Screen of a Regulatory *spx* Mutant Unveils a Role for the *ytpQ* Gene in the Control of Iron Homeostasis

**DOI:** 10.1371/journal.pone.0025066

**Published:** 2011-09-20

**Authors:** Peter Zuber, Shefali Chauhan, Praseeda Pilaka, Michiko M. Nakano, Sairam Gurumoorthy, Ann A. Lin, Skye M. Barendt, Bui Khanh Chi, Haike Antelmann, Ulrike Mäder

**Affiliations:** 1 Division of Environmental and Biomolecular Systems, Institute of Environmental Health, Oregon Health and Science University, Beaverton, Oregon, United States of America; 2 Institute for Microbiology, Ernst-Moritz-Arndt-University of Greifswald, Greifswald, Germany; 3 Interfaculty Institute for Genetics and Functional Genomics, Ernst-Moritz-Arndt-University of Greifswald, Greifswald, Germany; Baylor College of Medicine, United States of America

## Abstract

Spx is a global regulator of genes that are induced by disulfide stress in *Bacillus subtilis*. The regulon that it governs is comprised of over 120 genes based on microarray analysis, although it is not known how many of these are under direct Spx control. Most of the Spx-regulated genes (SRGs) are of unknown function, but many encode products that are conserved in low %GC Gram-positive bacteria. Using a gene-disruption library of *B. subtilis* genomic mutations, the SRGs were screened for phenotypes related to Spx-controlled activities, such as poor growth in minimal medium and sensitivity to methyglyoxal, but nearly all of the SRG mutations showed little if any phenotype. To uncover SRG function, the mutations were rescreened in an *spx* mutant background to determine which mutant SRG allele would enhance the *spx* mutant phenotype. One of the SRGs, *ytpQ* was the site of a mutation that, when combined with an *spx* null mutation, elevated the severity of the Spx mutant phenotype, as shown by reduced growth in a minimal medium and by hypersensitivity to methyglyoxal. The *ytpQ* mutant showed elevated oxidative protein damage when exposed to methylglyoxal, and reduced growth rate in liquid culture. Proteomic and transcriptomic data indicated that the *ytpQ* mutation caused the derepression of the Fur and PerR regulons of *B. subtilis*. Our study suggests that the *ytpQ* gene, encoding a conserved DUF1444 protein, functions directly or indirectly in iron homeostasis. The *ytpQ* mutant phenotype mimics that of a *fur* mutation, suggesting a condition of low cellular iron. In vitro transcription analysis indicated that Spx stimulates transcription from the *ytpPQR* operon within which the *ytpQ* gene resides. The work uncovers a link between Spx and control of iron homeostasis.

## Introduction

Transcriptome profiling can place genes into regulons or stimulons by providing evidence for coordinate control, governed by a transcriptional regulator and responsive to a specific metabolic or environmental stimulus [1]. In Gram-positive bacteria, some regulons, such as those controlled by alternative RNA polymerase sigma subunits [2] and global regulators [3] are large and complex. For example, the general stress SigmaB regulon, transcription of which requires the alternative RNA polymerase form bearing the σ^B^ subunit, is estimated to include over 200 genes in *L. monocytogenes* [4] and over 120 genes in *B. subtilis* [5], [6], [7], [8]. Many of the genes within complex regulons are of unknown function and the sites of mutations having no detectable phenotype. Hence our view of the roles global regulators play in bacterial physiology remains incomplete. We can imagine that the genes within these complex regulons reside in functionally redundant or genetically buffered subgroups required for alleviating stress by detoxifying or removing harmful agents, or repairing the damage such agents inflict upon macromolecules and supramolecular structure.

The Spx protein is a global regulator of the Gram-positive bacterium's stress response [9]. It is highly conserved in low G+C Gram-positive bacteria [10], and in *B. subtilis* it interacts with RNA polymerase to exert positive and negative transcriptional control over a genome-wide scale [11], [12], [13]. The products of genes having known function that are induced by Spx include thioredoxin, thioredoxin reductase, and products that function in cysteine biosynthesis as well as synthesis of the low molecular weight redox buffer, bacillithiol [12], [14], [15]. Spx activates the transcription of its regulon in response to disulfide stress and in cells treated with various toxic agents including paraquat, nitric oxide, cell wall-acting agents, toxic electrophiles and hypochloric acid [12], [16], [17], [18], [19], [20], [21]. Spx is under tight regulation that involves positive and negative transcriptional control [22], [23], [24] and proteolytic control by a substrate-binding factor, YjbH, together with the ATP-dependent protease, ClpXP [25], [26], [27], [28]. Additionally, its activity is controlled by a disulfide redox switch involving a CXXC motif at the N-terminal end of Spx that affects the protein's productive interaction with RNA polymerase [11]. Spx governs a large regulon with about 120 of its members designated as “y” genes of unknown function (Table S1). Because the Spx regulon is induced under a variety of stress conditions, uncovering the function of the Spx-regulated genes (SRGs) would further define the role of Spx in the cell's response to encounters with harmful agents.

In recent years, methods of genetic analysis have been developed to exploit the vast collections of genomic data generated from whole genome sequencing projects. Large gene knock-out libraries have been created and utilized to uncover functional genetic modules consisting of genes that influence essential cellular processes. One way this has been accomplished is by the systematic and automated screening of strains with paired mutations (double mutants) to search for synthetic phenotypes indicative of genetic interaction [29], [30], [31], [32], [33], [34], [35]. The rationale for uncovering modules of interacting genes has its origins in concepts of functional redundancy and genetic buffering [36]. Elegant studies using classic genetic systems, and screens for unlinked non-complementation and synthetic lethality, uncovered genes that reside within functional modules that affect, for example, morphogenesis and the dynamics of cytoskeletal components [37], [38], [39], [40], [41]. More recent studies of genome-wide synthetic genetic arrays uncovered new factors involved in iron metabolism and in the activity of the transcription complex [29]. Thus genetic screens for synthetic interaction and phenotype enhancement can shed light on the functions of genes for which no known function has been assigned. Hence, we undertook a phenotype enhancement screen of *spx* mutants bearing knock-outs of each of the SRGs of unknown function. Strains with the *spx* mutation paired with each *srg* mutation that have defects in growth or elevated sensitivity to methylglyoxal, to which *spx* mutants are sensitive, were detected. One such *srg* mutation, in the *ytpQ* gene, was studied using proteomic and transcriptomic analyses, which uncovered a role for *ytpQ* in iron metabolism/homeostasis.

## Results and Discussion

Results of previously published microarray hybridization data [12], uncovered about 125 genes of the “y” designation that were more than 3-fold upregulated by high Spx concentrations (Table S1). The majority of the genes are of unknown function, although many encode products that are highly conserved in other, mostly Gram-positive, bacterial species. While we do not know at this point if all of the genes are under the direct transcriptional control of Spx, we decided to designate the genes as SRGs, or Spx-regulated genes.

The *spx* regulon is induced by a number of toxic agents and stress conditions, [10], [12], [16], [17], [20], [22], [42]. To understand further the role of Spx in the cell's response to these diverse stress conditions, we sought to gain information on the function of the individual SRGs. A *B. subtilis* ORF knock-out library, obtained from K. Kobayashi and N. Ogasawara (NAIST, Japan), contains gene disruptions in over 2,000 ORFs that are assigned the “y” genomic designation [43]. Each disruption was created by an integrated DNA fragment, derived from plasmid pMUTIN [43], that was inserted within the target gene's coding sequence. The fragment contains a promoter-less *lacZ* gene at its 5′end, followed by the *E. coli lacI* gene, the product of which controls an IPTG-inducible P*_spac_* promoter [44] residing at the 3′ end of the fragment and oriented in the 3′ direction. The P*_spac_* promoter in this position can drive expression of genes located downstream of the insertion [43], thus alleviating potential insertion-dependent polar effects. The use of pMUTIN-mediated gene disruption was used in previous genome-wide mutational screens and in gene interaction studies [45], [46]. Gene disruptions in the SRGs listed in Table S1 were tested by screening for growth on nutrient broth sporulation medium (DSM) and minimal glucose (TSS) medium plates. Some slight defect in growth on TSS agar plates was detected for some of the SRG mutants, but the majority showed no obvious defect in growth based on colony size. We next tested the SRG mutant strains for defects in growth on DSM agar plates containing methylglyoxal (MG), a toxic alpha-oxoaldehyde to which *spx* mutants are sensitive (data not shown). Again, we observed only minor growth defects compared with the wild-type parent on DSM agar medium containing concentrations of methylglyoxal to which *spx* mutants are sensitive.

### Phenotype enhancement screen of *srg* mutations in the *spx* mutant background

**Figure 1:**
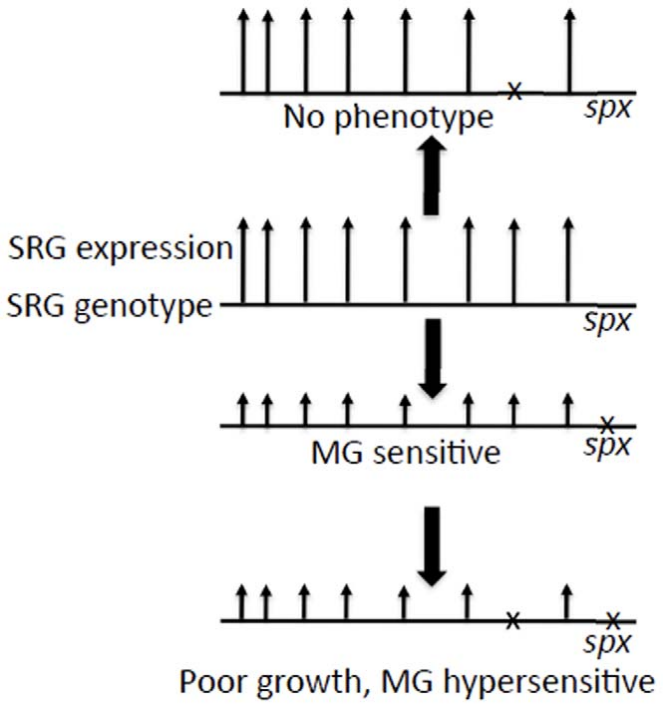
Rationale for phenotype enhancement screen. Horizontal line denotes genotype and vertical arrows represent level of expression of each gene. SRG is Spx-regulated gene. The X on the horizontal line is a null mutation, eliminating expression. The SRG mutation yields little observable phenotype with respect to electrophile stress. The *spx* mutation reduces overall SRG expression, thus reducing effect of genetic buffering and functional redundancy. The *srg spx* double mutant is now found to be hypersensitive to electrophile (methylgyoxal), or shows other defects such as reduced growth rate.

Possible reasons for the absence of SRG mutant phenotype related to stress resistance include genetic interactions built into the *spx* regulon that contribute to functional redundancy and genetic buffering. We reasoned that uncovering phenotype would require overcoming regulon genetic interactions that mask defects conferred by the SRG mutations. Hence, we undertook a screen of the SRG mutations within the *spx* deletion mutant background. The reasoning is illustrated in Figure 1, where the *spx* mutation is shown to cause a reduction in overall SRG expression, thereby reducing the contributions of genetic interactions among SRGs. Introduction of the *srg* mutation into the *spx* mutant background potentially confers hypersensitivity to the agents to which the *spx* mutant is sensitive, but the *srg spx* mutant would now be predicted to exhibit further reduction in growth on minimal medium, and hypersensitivity to lower toxic agent concentrations that have little to no effect on the parent *spx* mutant.

Each plate was inoculated with 10 µl of a dilution series of a log phase culture. The size of the isolated colonies was then measured using the Pixicillus application (Material and Methods). An example of a plate and data collected is shown in Figure S1, where four strains, the wild type JH642, the *spx* mutant, the SRG mutant, *yitV*, and the *spx yitV* double mutant plated on DSM and DSM plus MG is shown. The average isolated colony size as determined by Pixicillus indicated that the double mutant has a slight growth defect on the DSM-MG plate compared with the *spx* mutant parent. Most of the double mutants screened showed a result similar to that uncovered in the *yitV spx* mutant.

### Disruption of *ytpQ*, an Spx-regulated gene of unknown function, enhances *spx* mutant phenotype when combined with an *spx* null mutation

**Figure 2:**
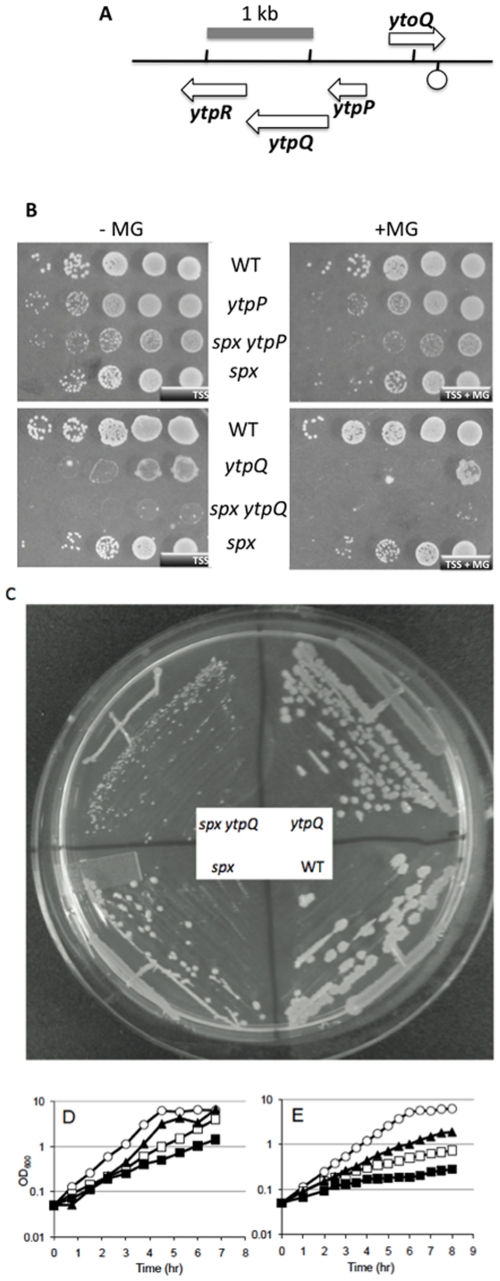
Phenotype of *srg* mutations in *spx* mutant background. A. Chromosomal organization of *ytoQ ytpQ* locus. Arrows represent location and orientation of coding sequences and lollipop figure denotes location of putative transcriptional terminator. B. Phenotype of *ytpP* and *ytpQ* mutations in the wild-type and *spx* mutant backgrounds. Cultures were grown to late log phase and serially diluted 10-fold. Ten µl were spotted on TSS minimal medium plates with and without MG. C. Minimal TSS plate onto which JH642 (wild-type parent), the *spx* mutant, the *ytpQ*::pMUTIN mutant, and the double mutant *spx ytpQ*::pMUTIN were streaked. D and E. Growth curves of *ytoQ* and *ytpQ* mutants in wild-type and *spx* mutant backgrounds. Open circles: JH642. Open squares: *spx* mutant. Closed triangles *ytoQ* mutant (D), and *ytpQ* mutant (E). Closed squares: *ytoQ spx* mutant (D) and *ytpQ spx* mutant (E).

Other double mutants showed more dramatic phenotype enhancement on TSS agar plates than was observed in the case of the *yitV* mutant. The linked genes *ytoQ* and *ytpQ* (Figure 2A) were the sites of two gene disruptions that showed growth defects in the *spx* mutant background. The *ytpQ spx* mutant showed a severe growth defect on TSS minimal medium (Figure 2B and 2C). The phenotypes of the *ytoQ spx* and *ytpQ spx* mutants were examined in growth curves of cultures in liquid TSS medium (Figure 2D and E). Both *ytpQ* and *ytoQ* mutants and the *spx* null mutant showed defects in growth as evident in the slope of the log phase portion of the growth curves or final OD_600_ (doubling times: JH642; 42.8 min. *spx* null mutant; 93.8 min. *ytoQ*; 60.3 min. *ytpQ*; 73.3 min). The double mutants showed a further reduction in growth rate (*ytoQ spx*; 103.8 min, *ytpQ spx*; 164.9 min) and had a lower growth yield. While the introduction of the *ytpQ* mutation into the *spx* null mutant resulted in a slower growth rate than the *spx* null, the doubling time of the *ytoQ spx* strain was not significantly longer than that of the *spx* null. Hence, we did not further analyze the *ytoQ* mutant.

The *ytpQ* and *ytoQ* genes are linked and divergently oriented in the *B. subtilis* chromosome (Figure 2A). The *ytpQ* gene product is a member of the DUF1444 family of bacterial proteins with no known function. The *ytpQ* gene is part of a tricistronic operon that also contains *ytpP* and *ytpR*. Disruption of *ytpR* showed no noticeable phenotype on DSM or TSS medium with or without MG, and confers no phenotype enhancement in the *spx* mutant background (data not shown). The disruption of the *ytpP* gene, encoding a thioredoxin-like protein [47], conferred phenotype enhancement in the *spx* background (Figure 2B), but this was reversed by addition of IPTG, showing that the defect was due to a polar effect of the *ytpP* insertion (data not shown), most likely causing reduction in *ytpQ* gene expression.

Complementation was conducted using an IPTG-inducible version of the *ytpQ* gene ectopically expressed from the *amyE* locus of the *B. subtilis* genome. For these experiments, a deletion *ytpQ* mutation was constructed in which part of the *ytpQ* coding sequence was replaced by a spectinomycin-resistance cassette. An *spx ytpQ* double mutant (ORB7816) was then constructed by introducing the *ytpQ*::*spc* mutation into a *spx*::*tet* (tetracycline resistance) mutant. As was observed with the *ytpQ*::pMUTIN *spx* double mutant, the ORB7816 strain enhanced the growth-defective phenotype compared with either the *ytpQ* or *spx* mutants in the presence or absence of MG (Figure S2). Thus, a strain bearing the new *ytpQ*::*spc* allele and the ectopic inducible *ytpQ* construct was grown in TSS minimal medium in the presence and absence of 0.5 mM IPTG. The results (Figure S3) showed that the reduced growth rate of the *ytpQ* mutant (doubling time of 79.4 min) was reversed when the ectopically expressed *ytpQ* gene was induced. The induced complemented strain (ORB8011) had a growth rate similar to the wild type parent (JH642; doubling time of 47.3 min. OR8011; doubling time of 46.5 min).

### The *ytpPQR* operon is under direct Spx control

**Figure 3:**
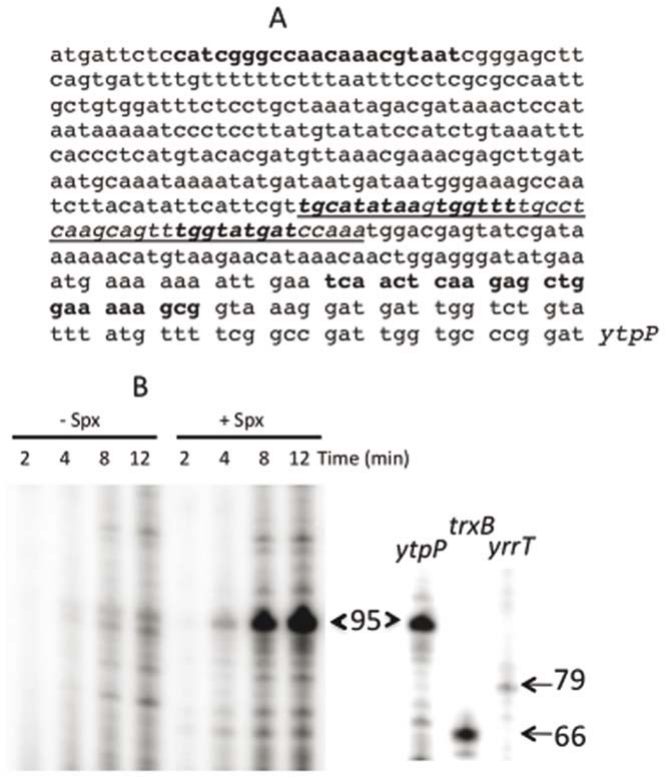
Spx-activated transcription from the *ytpPQR* operon promoter. A. The nucleotide sequence of the *ytpPQR* promoter region is shown, The bold plain text indicates the oligonucleotide primers used to generate the linear DNA promoter template fragment for the in vitro transcription reaction. Also the region underlined and in italics shows the putative promoter region along (in bold) the −10 region (***tatgat***) with extended TG and −35 region (***tggttt***) and the Spx cis element (***tgcatataa***) [77] upstream from the −35 region. B. In vitro transcription from *ytpP* promoter. The *ytpP* promoter template (10 nM) was incubated with 25 nM σ^A^-depleted RNAP and 25 nM σ^A^ in the absence or presence of 75 nM Spx. Samples were collected from the reactions at indicated times during incubation (2, 4, 8, and 12 min). Transcripts were resolved by gel electrophoresis, visualized and quantified as previously described [77]. Marker transcripts were generated using Spx protein and purified RNA polymerase, along with DNA fragments containing the Spx-controlled *trxB* promoter [11] and the *yrrT* promoter [14] in transcription reactions.

To validate the previous microarray results, the *ytoQ* and *ytpQ* pMUTIN insertions, both generating transcriptional *lacZ* fusions, were used to measure *ytoQ*- and *ytpQ*-directed β-galactosidase activity in cells expressing an IPTG-inducible, protease resistant form of Spx (Spx^DD^) that were grown in liquid DSM medium (Figure S4). In keeping with the microarray results, the fusions showed elevated expression when the *spx^DD^* allele was induced. Furthermore, microarray analysis (described below) indicated reduced *ytpQ* transcript levels in the *spx* mutant (Table S2). Validation was also accomplished by transcription analysis performed in vitro, which showed that addition of Spx in a reaction with the *ytpPQR* promoter region DNA and RNA polymerase resulted in the synthesis of a transcript of the predicted size (Figure 3). Synthesis of the transcript is stimulated by the addition of Spx and initiates near a σ^A^-recognized promoter sequence (Figure 3A and B). The reaction utilized His-tagged RNA polymerase from an *rpoDL366A* mutant from which RNA polymerase lacking σ^A^ was obtained. Purified σ^A^ was required for each reaction in order to observe a transcript from the *ytpP* promoter template (data not shown). This result is consistent with the presence of a σ^A^-utilized promoter upstream of the *ytpP* coding sequence.

### The *ytpQ* mutant has increased levels of protein damage after MG treatment

**Figure 4:**
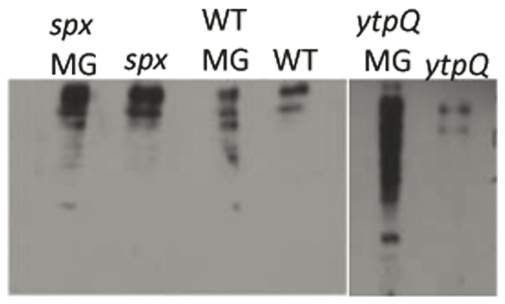
Assay of protein damage in wild-type, *spx*, and *ytpQ* strains in the presence and absence of methylglyoxal (MG). Cells were incubated in 100 ml 2xYT cultures to an OD_600_ of 0.6. Cultures were split and MG to 2 mM was added to one of the two cultures. Crude cell extracts were prepared for SDS-PAGE and oxyblot analysis was performed as described in Materials and Methods. The same amounts of protein were applied to the SDS-PAGE gel. WT denotes blot of JH642 culture cell extracts. MG – methylglyoxal treatment.

We further examined the phenotype of the *ytpQ* mutant in order to gain more information about its possible role in the *B. subtilis* stress response. Previous microarray hybridization studies indicated that *ytpQ* was derepressed in a *perR* [peroxide regulator [48]] mutant background [49]. That *ytpQ* is derepressed in a *perR* mutant and activated by Spx suggests that its product might function in the oxidative/electrophile stress response. We determined if the *ytpQ* mutant shows elevated levels of protein carbonylation damage by performing an oxyblot [50] on cell extracts from cultures of JH642, *spx* mutant, and *ytpQ*::*spc* mutant cells that were untreated or treated with MG. The untreated wild-type cells showed some evidence of oxidative protein damage (Figure 4), which was increased upon MG treatment. The untreated *spx* mutant cells showed more damage than the wild type parent, with some increase in damage following MG treatment. The untreated *ytpQ* mutant cells resembled the untreated wild-type parent in the level of oxidatively damaged protein, but the mutant underwent a dramatic increase in the level of protein damage upon MG treatment that was much higher than the treated wild-type parent or *spx* mutant. The result suggests that the *ytpQ* product plays a role in preventing protein damage resulting from an encounter with a toxic electrophile (MG).

### Transcriptomic and proteomic analyses indicate a role of *ytpQ* in iron homeostasis

**Figure 5:**
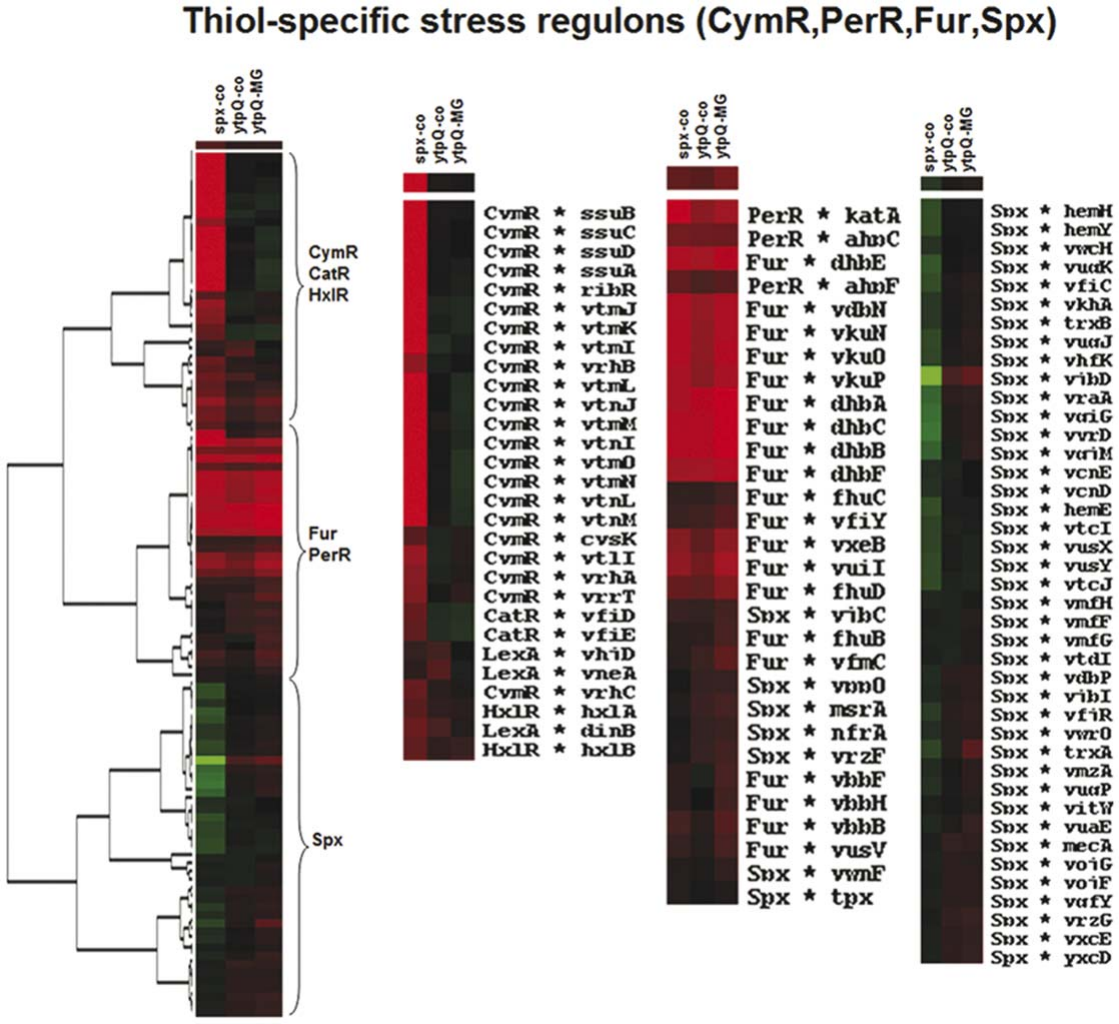
Hierarchical clustering analysis of gene expression profiles up- and downregulated in *spx* and *ytpQ* mutants. Gene expression data were clustered based on the induction or repression ratios in the *spx* and *ytpQ* mutants leading to different nodes specific to regulons. Nodes enriched for genes that belong to the thiol-specific stress regulons (CymR, Spx, PerR, Fur, HxlR, CatR) are shown.

**Figure 6:**
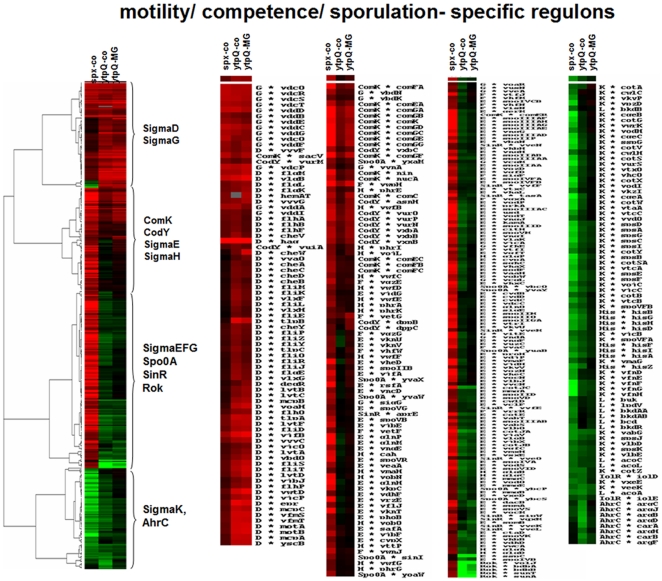
Hierarchical clustering analysis of gene expression profiles up- and downregulated in *spx* and *ytpQ* mutants. Gene expression data were clustered based on the induction or repression ratios in the *spx* and *ytpQ* mutants leading to different nodes specific to regulons. Nodes including regulons involved in motility, competence and sporulation are shown (Com, SigmaD, SigmaH, CodY, SinR, Spo0A, SigmaL, AhrC, Rok, SigmaE, F, G, K regulons). Red indicates induction and green repression in the *spx* or *ytpQ* mutants under control conditions, and MG stress.

To gain more insight into the function of *ytpQ*, the transcriptome of the y*tpQ* mutant was analyzed, and compared with that of the wild type and the *spx* mutant. Similarities in the transcriptomic changes conferred by the *spx* and *ytpQ* mutations would provide clues to the role played by YtpQ within the Spx regulon. The wild-type parent, *ytpQ* and *spx* mutants were grown in a glucose minimal medium, with and without 2.8 mM MG, to mid-log phase. Cells were harvested and RNA was extracted for microarray hybridization analysis to determine if any change could be detected in the composition of the transcriptome that were attributable to the *ytpQ* mutation (data in Tables S2 and S3). Previous microarray hybridization analysis identified putative Spx-controlled genes by detecting elevations in transcript levels when protease resistant forms of Spx were produced [12]. The transcriptome was reexamined, this time, by conducting microarray analysis with an *spx* null mutant. As predicted from the previous work, the *spx* mutation causes extreme changes to the cell transcriptome [12], [14], [27], [51]. There is a dramatic increase in the level of transcripts from CymR-controlled genes, whose products function in organosulfur metabolism and the synthesis of cysteine (Figure 5, Table S2) [14], [52], [53]. In fact, the impaired growth of the *spx* mutant on minimal medium can be reversed by addition of cysteine, indicating that the *spx* mutant is a mild Cys auxotroph. The reduced expression of the *trxA* gene might account for *spx* growth phenotype on minimal medium, since thioredoxin is required for sulfate utilization [54]. There is an increase in the level of transcript from genes controlled by PerR and Fur, both Fe-binding proteins that regulate, respectively, the peroxide response and genes that are activated under iron starvation (Figure 5, Table S2). ComK-dependent transcription was also derepressed confirming previously reported results [55], [56]. Synthesis of transcripts encoded by early sporulation genes controlled by σ^H^, σ^E^, and σ^F^ was also up-regulated in the *spx* mutant background. ClpX was known to be required for σ^H^-dependent transcription (Figure 6, Table S2) [57], [58], which was partially relieved by a mutation in the *spx* gene [55].

The most striking result from the transcriptome analysis of the *ytpQ* mutant is the dramatic derepression of the Fur (iron uptake regulator) and PerR (peroxide response regulator) regulons (Figure 5, Table S2 and S3). The *ytpQ* mutation seems to mimic the reported phenotype of the *B. subtilis fur* mutant [59]. The expression of genes (*dhbABCDEF*) encoding the enzyme complex that catalyzes dihydroxybenzoyl-glycine (DHB-Gly, or itoic acid) synthesis was dramatically increased. The *B. subtilis* strain, JH642, bears a mutation in the *sfp* gene [60] encoding phosphopantetheinyl transferase [61] required for non-ribosomal peptide synthetase activity. Hence, JH642 cells are unable to synthesize the siderophore, bacillibactin (DHB-Gly-Thr) [62] despite the fact that genes required for its biosynthesis (the *dhb* operon) show elevated expression. The genes specifying hydroxamate siderophore utilization (*yxeB*, *fhuB*, *fhuD*) were also upregulated in the *ytpQ* mutant. The expression of the Fur-regulated *ykuNOP* operon was elevated higher than 10-fold in the *ytpQ* mutant. The operon encodes two flavodoxins that serve as reductases for nitric oxide synthase catalysis and for two-electron transfer to cytochrome P450 BioI. This latter function can be fulfilled by the product of the *fer* gene, which is a 4Fe-4S ferridoxin. Under conditions of low iron, the *ykuN*, and *ykuP* products provide an iron-free substitute. Another characteristic of the *ytpQ* mutant that mimics the *fur* null phenoytpe is the derepression of the genes belonging to the cryptic prophage, PBSX (Figure S5) [59]. It is not known why these genes show elevated expression in the *fur* mutant.

Notably, several of the genes derepressed in the *ytpQ* mutant were further up-regulated upon MG treatment (Figure 5, Tables S2, S3). These include genes controlled by PerR and Fur, as well as the *spx* gene itself. Elevated expression of genes encoding the transporter for elemental iron (*yfmLMN*) was observed in the *ytpQ* mutant cells treated with MG.

Another class of genes showing elevated expression in the *spx* and *ytpQ* mutant is the σ^D^ regulon, which includes genes that function in motility and chemotaxis [63], [64]. The level of the *hag* gene transcript, encoding flagellin, was increased 80-fold in the *ytpQ* mutant (Figure 6). It is not clear why the expression of the σ^D^ regulon is elevated in the *ytpQ* mutant. Reduced growth rate of the *ytpQ* mutant could be related to the elevated expression of genes that function in motility and chemotaxis. Possibly linked to this phenotype is the elevated expression of *ywaC* in the *ytpQ* mutant (3.5-fold). The *ywaC* gene product is a GTP pyrophosphokinase that can catalyze ppGpp formation at the expense of GTP [65], [66]. Induction of the stringent response has been observed to heighten expression of the motility/chemotaxis regulons through reduced activity of the CodY repressor [67]. Another report has linked iron-dependent control and CodY with the stringent response [68].

**Figure 7:**
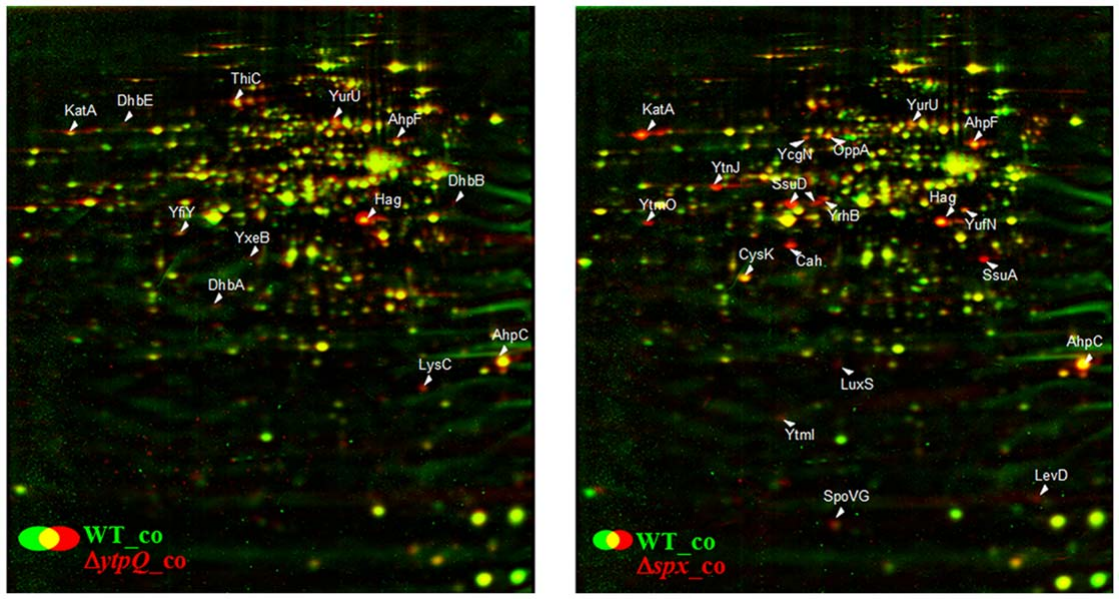
Dual-channel images of the protein amounts in *B. subtilis* wild type (green image) compared to the *ytpQ* (left) and *spx* mutants (right) (red images) under control conditions. Cytoplasmic proteins were separated by 2D PAGE as described in Materials and Methods. Image analysis was performed using the Decodon Delta 2D software. Proteins with increased levels in the mutants in at least two independent experiments are indicated by white labels.

2D-gel based cytoplasmic proteome analysis provides validation of the transcriptomic results (Figure 7). Consistent with the microarray data of the *spx* mutant, the most strongly up-regulated proteins in the proteome are controlled by the CymR repressor. These include YtmI, YtmO, YtnJ, YrhB, CysK, SsuA, SsuD. Furthermore, the PerR-controlled proteins KatA, AhpC, AhpF were strongly elevated in the *spx* mutant proteome and less strongly in the proteome of the *ytpQ* mutant. The proteins of the Fur-regulated genes *dhbA*, *dhbB*, and *dhbE* were also observed at increased amounts in the *ytpQ* mutant. Finally, the Hag protein amount was also elevated in mutant cells. The elevated protein levels in the *ytpQ* mutant are likely the result of the enhanced transcription uncovered in the transcriptome analysis.

The phenotype enhancement screen is one way to gain information about SRG function. In the present study, Spx phenotype enhancement was tested by examining growth on minimal medium and sensitivity to methylglyoxal. However, the Spx regulon is induced under a variety of harsh conditions, and an *spx* null mutant is sensitive to other toxic agents including electrophiles, paraquat, selenite, hypochlorite and certain antibiotics (unpublished data). Resistance to each of these agents might require the contributions of one or more specific SRGs. With this in mind, further phenotype enhancement screens can be conducted in the presence of each toxic agent with SRG mutations in the *spx* mutant background to initiate an effort to uncover function of other Spx regulon members.

The *spx* phenotype enhancement screen of SRGs uncovered the *ytpQ* gene as being an important member of the *spx* regulon. The double *spx ytpQ* mutant exhibited severely impaired growth in minimal medium. Phenotype analysis of the *ytpQ* mutant showed that it had an elevated level of oxidative protein damage after methylglyoxal treatment compared to that of the wild-type parent. The increased protein damage upon MG treatment could be a consequence of dysfunctional iron metabolism, which was evident from the transcriptome results showing heightened expression of Fur regulon genes in *ytpQ* mutant cells. While phenotype of the *ytpQ* mutant resembled that of a *fur* mutant, indicating a condition of low cellular iron, this also creates a condition of elevated free, chelatable iron that could mediate the observed oxidative damage [69], [70](Figure 4). These results implicate *ytpQ* gene as a link between Spx-dependent control and regulation of iron homeostasis. The study herein provides evidence that the influence of Spx in oxidative stress management extends to participation in the control of iron metabolism. Hence, the severity of the *spx* phenotype is enhanced by the loss of *ytpQ* function, and the accompanying dysfunction in iron homeostatic mechanisms.

Attempts at gaining more information about YtpQ function will involve a search for interacting proteins, with hopes of finding binding partners with known function. Screening other SRG mutations in the *ytpQ* background to find synthetic effects or phenotype enhancement will uncover other *spx* regulon members that reside in the same functional domain occupied by *ytpQ*. Suppressor mutations that relieve the growth impairment of the *ytpQ spx* mutant will also identify genes that are influenced by YtpQ function.

## Methods

### Bacterial strains and growth media

The *B. subtilis* knock-out library [45] was constructed using a previously described method [43], and was obtained from the laboratory of N. Ogasawara (NAIST, Nara, Japan). Mutants from the collection were re-checked by PCR using primers that hybridize to the genomic region where the disruptions reside and primers specific to pMUTIN. This was done to ensure that the pMUTIN insertion was in the assigned gene. DNA from the SRG members (Table S1) of the mutant collection was used to transform JH642 and ORB6781 (*spx*::*spc*) competent cells to create two sets of isogenic SRG mutants. All strains used in this study were derived from JH642 (*trpC2 pheA*) and are Trp^−^ and Phe^−^ auxotrophs.

*B. subtilis* cells were grown on TSS minimal medium [71], 2xYT (yeast extract/tryptone [72]), or DSM nutrient broth sporulation medium [73]. *E. coli* plasmid-bearing strains were propagated in 2xYT. Antibiotic concentrations used in growth media were ampicillin (50 µg ml^−1^), chloramphenicol (5 µg ml^−1^), spectinomycin (75 µg ml^−1^), and erythromycin/lincomycin (1 µg ml^−1^ and 25 µg ml^−1^, respectively).

### Construction of *ytpQ* deletion mutant

The *ytpQ* gene was amplified from JH642 chromosomal DNA using a forward primer oMN10-505 (cgCCGCAAAACAAAAGAAGAA; only upper case letters correspond to chromosomal sequence) and a reverse primer oMN10-506 (cgCCACACCTTCTTTATTATA) by PCR. The amplified DNA was cloned using Topo-cloning kit (Invitrogen) to generate pMMN806. The *ytp*Q gene isolated from EcoRI-digested pMMN806 was cloned into pUC8 digested with EcoRI to generate pMMN807. The spectinomycin-resistant cassette was isolated from pDG1727 digested with BamHI and StuI and the resistance gene was used to replace the BglII/EcoRV-digested fragment of *ytpQ* in pMMN807. The resultant plasmid pMMN808 led to a substitution of a 54-bp DNA in *ytpQ* with the spectinomycin-resistance gene. The plasmid pMMN808 was used to transform JH642 and the transformant (ORB7815) was selected for the spectinomycin resistance. The disruption of *ytpQ* in ORB7815 was confirmed by PCR using oMN10-505 and oMN10-506. ORB7816 (*spx*::*tet ytpQ*::*spc*) was constructed by transforming ORB6876 (*spx*::*tet*) with chromosomal DNA prepared from ORB7815.

### Preparation of chromosomal DNA and transformation

Small-scale chromosomal DNA preparation was conducted by growing a culture of 3 ml 2xYT. The cells were harvested at 14,000 rpm in a Sorvall super T21 centrifuge with SL-50T rotor at 4°C for 10 min. The cells were resuspended in EDTA buffer (25 mM NaCL, 50 mM EDTA). Lysozyme was added and the mixture was then incubated for 15 min. Later sarkosyl was added and phenol/chloroform extraction was performed. The chromosomal DNA was then stored at 4°C. The chromosomal DNA from the mutants was used to transform JH642 competent cells and *spx* mutant (ORB6781) competent cells. The transformation mixture was incubated for 30 min at 37°C and then 1 ml of 2xYT was added in each test tube. The transformation mixture was incubated for 1 h and plated on DSM Erm-Ln (1 µg/ml erythromycin and 25 µg/ml lincomycin) and DSM Spc (75 µg/ml spectinomycin) plates, respectively. The colonies were then streaked for single cell clones. After genotype testing, strains were stored at −80°C.

### Mutant Screening

The SRG mutations in the wild-type and *spx* mutant backgrounds were tested on TSS, TSS+IPTG (0.5 mM), TSS+MG (2 mM), TSS+MG+IPTG plates and the phenotype of each strain was recorded. The mutants in both backgrounds along with JH642 and *spx*::*spc* were grown in TSS media with appropriate antibiotics. The cells were diluted to OD_600_ = 0.1 and then serially diluted to 10^−6^ in TSS medium. Ten µl from each dilution were transferred to TSS plates and TSS and MG (2 mM) plates.

To assess the extent of growth impairment on minimal medium or caused by treatment with methylglyoxal, we employed a Matlab graphical user interface to measure colony size on agar medium containing the toxic agent. The application, Pixicillus, utilizes triangulation of coordinates corresponding to the dimensions of the petri plate as a standard to calculate colony size (the design and use of the program is available upon request).

### Construction of *ytpQ* complementation strains

The *ytpQ* ORF as well as 37 bases upstream harboring the *ytpQ* Shine-Dalgarno sequence was amplified by PCR from JH642 purified genomic DNA using primers oSB1 (TAGGGAAGCTTCAGACTCTCTCGCTAAAGCGTAAAGGA, HindIII cut site underlined) and oSB2 (TAGGCTCTAGACTAATCCTTTTTCGGACGGCTTTTCGC, XbaI cut site underlined). The HindIII - XbaI digested linear amplicon was cloned into pDR67 [74], which contains an IPTG-inducible *spac* promoter [44], a chloramphenicol resistance cassette, and flanking *amyE* homologous regions for chromosome integration. pDR67::P*spac*-(SD*ytpQ*)-*ytpQ* (pSB1) was sequenced and introduced by transformation into several competent isogenic strains of *B. subtilis*: i) *spx*::*tet* (ORB6876), ii) *ytpQ*::*spc* (ORB7815), iii) *spx*::*tet ytpQ*::*spc* (ORB7816), and iv) parental JH642. Strains were tested for the disruption of the *amyE* locus by plating on LB plus 0.5% starch.

### Growth curves

The preculture of JH642, *spx*::*spc* and mutants constructed in the JH642 or *spx*::*spc* genetic backgrounds were grown in TSS media with appropriate antibiotics until mid-log phase. The cells were then diluted to OD600 = .03 and incubation was continued with shaking at 37°C. The OD_600_ was taken at time intervals of 30 min or 1 h.

### OxyBlot Assay

The wild-type strain, *ytpQ* mutant, and *spx*::*spc* mutant were grown in 100 ml of 2xYT at 37°c. At OD_600_ = 0.5, the culture was divided equally in baffled flasks for each sample. Methylgloxal (2.8 mM) was added into one of the two flasks. The cells were grown for 6 h and harvested at 7000 rpm, 4°C for 20 min in a Sorvall Super T21 centrifuge using a SL-50T rotor. The harvested cells were resuspended in 50 mM NaCl, 25 mM EDTA pH 7.0 and lysed using a French press. The crude cell lysate was centrifuged as before. The supernatant was collected and the protein concentration was determined by the Bradford Assay [75]. Fifteen µg of the crude sample protein was derivatized with 2, 4 dinitrophenyl hydrazine. A control reaction with extract of untreated cells was also assembled. The samples were then applied to a 12% SDS polyacrylamide gel and the protein was electroblotted onto a nitrocellulose membrane. The membrane was treated with primary antibody provided in the Oxyblot kit (Millipore) specific for 2,4 dinitrophenol hydrazone. After the treatment with antibodies the membrane was treated with chemi-luminescent reagent (Millipore) and labeled protein was visualized on a photographic film (Fuji).

### In vitro transcription

RNA polymerase and Spx protein was purified as previously described [28], [76]. For the study reported herein, RNA polymerase was purified from an *rpoDL366A* mutant (A gift from C. P. Moran, Jr., Emory University), which lacks SigA protein after a three-column purification procedure [76]. The enzyme does not transcribe a consensus σ^A^ promoter DNA fragment (from the *rpsD* gene) unless purified σ^A^ [58] is added to the reaction. In vitro transcription reactions were performed according to previously published work [11], [76], [77], with further details in Figure legend 3.

### Proteome and mass spectrometry analysis

*B. subtilis* wild type (JH642), *ytpQ* and *spx* mutant cells were grown in Belitsky minimal medium [78] to an OD_500_ = 0.4 and harvested before (control) and 20 min after exposure to 2.8 mM methylglyoxal. Preparation of cytoplasmic protein extracts and separation by two-dimensional gel electrophoresis (2D-PAGE) were performed as described [79]. The protein content was determined using the Bradford assay [75]. For two-dimensional gel electrophoresis (2D-PAGE), 200 µg of the protein extracts were separated using the non-linear immobilized pH gradients (IPG) in the pH range 4–7 for cytoplasmic proteins (Amersham Biosciences) and a Multiphor II apparatus (Amersham Pharmacia Biotech) as described previously [80]. The resulting 2D gels were fixed in 40% (v/v) ethanol, 10% (v/v) acidic acid and stained with Colloidal Coomassie Brilliant Blue (Amersham Biosciences). The image analysis was performed from the Coomassie-stained 2D gels using the DECODON Delta 2D software (http://www.decodon.com).

For protein identification from 2D gels, spot-cutting, tryptic digestion of the proteins, and spotting of the resulting peptides onto the MALDI-targets (Voyager DE-STR, PerSeptive Biosystems) were performed using the Ettan Spot Handling Workstation (Amersham-Biosciences, Uppsala, Sweden) as described previously [81]. The MALDI-TOF-TOF measurement of spotted peptide solutions was carried out on a Proteome-Analyzer 4800 (Applied Biosystems, Foster City, CA, USA) as described previously [81].

### Transcriptome analysis

For microarray analysis, *B. subtilis* wild type, *ytpQ* and *spx* mutant cells were grown in Belitsky minimal medium to OD_500_ of 0.4 and harvested before and after exposure to 2.8 mM methylglyoxal. Total RNA was isolated by the acid phenol method as described [82]. For transcriptome analysis, 35 µg RNA were DNase-treated using the RNase-Free DNase Set (Qiagen) and purified using the RNA Clean-Up and Concentration Micro Kit (Norgen). The quality of the RNA preparations was assessed by means of the Agilent 2100 Bioanalyzer according to the manufacturer's instructions.

Synthesis and purification of fluorescently labeled cDNA were carried out according to Charbonnier *et al.* [83] with minor modifications. In detail, 10 µg of total RNA were mixed with random primers (Promega) and spike-ins (Two-Color RNA Spike-In Kit, Agilent Technologies). The RNA/primer mixture was incubated at 70°C for 10 min followed by 5 min incubation on ice. Then, the following reagents were added: 10 µl of 5× First Strand Buffer (Invitrogen), 5 µl of 0.1 M DTT (Invitrogen), 0.5 µl of a dNTP mix (10 mM dATP, dGTP, and dTTP, 2.5 mM dCTP), 1.25 µl of Cy3-dCTP or Cy5-dCTP (GE Healthcare) and 2 µl of SuperScript II reverse transcriptase (Invitrogen). The reaction mixture was incubated at 42°C for 60 min and then heated to 70°C for 10 min. After 5 min on ice, the RNA was degraded by incubation with 2 units of RNaseH (Invitrogen) at room temperature for 30 min. Labeled cDNA was then purified using the CyScribe GFX Purification Kit (GE Healthcare). Five hundred ng of Cy5-labeled cDNA and 500 ng of Cy3-labeled cDNA were hybridized together to the microarray following Agilent's hybridization, washing and scanning protocol (Two-Color Microarray-based Gene Expression Analysis, version 5.5).

Data were extracted and processed using the Feature Extraction software (version 10.5, Agilent Technologies). For each gene on a microarray, the error-weighted average of the log ratio values of the individual probes was calculated using the Rosetta Resolver software (version 7.2.1, Rosetta Biosoftware). Genes showing induction or repression ratios of at least three-fold in three independent experiments were considered as significantly induced. The averages ratios and standard deviations for all induced or repressed genes in the *ytpQ* and *spx* mutants compared to the wild type were calculated from three independent transcriptome experiments each and listed in Table S3. All microarray datasets and accompanying descriptions are MIAME compliant and the datasets are available in the GEO database under accession numbers GSE28872.

### Hierarchical clustering analysis

Clustering of gene expression profiles up- and downregulated in *spx* and *ytpQ* mutants compared to the wild type under control conditions and in the *ytpQ* mutant under methylglyoxal stress were performed using Cluster 3.0 [84]. The transcriptome datasets included log2-fold expression changes in the *spx* and *ytpQ* mutant strains versus wild type. After hierarchical clustering, the output was visualized using TreeView [85]. For clustering, genes were used that are induced or repressed in the *ytpQ* and/or *spx* mutants (e.g. CymR, Spx, PerR, Fur, HxlR, CatR, Com, SigmaD, SigmaH, CodY, SinR, Spo0A, SigmaL, AhrC, Rok, SigmaE, F, G, K regulons and SPβ-related genes).

## Supporting Information

Figure S1 **Colonies of serially diluted, spotted cultures on TSS plates with and without 3 mM methylglyoxal.** Strains are JH642 (wild type parent), *spx* mutant, *yitV*, and *yitV spx* mutant. Values are average colony size as determined by Pixicillus.
(TIF)

Figure S2 **TSS minimal medium agar containing 0, 0.5, 1, and 2 mM MG.** From top to bottom rows: JH642 (Wild type parent), *spx*::*tet* mutant, *ytpQ* deletion mutant, *spx*::*tet ytpQ* deletion double mutant. Cells were grown in TSS medium to mid-log phase, then serially diluted 10-fold. Ten µl of each dilution was then spotted onto the agar surface. Plates were incubated at 37°C.
(TIF)

Figure S3 **Complementation of the Δ** ***ytpQ*** **::** ***spc*** **mutation by an IPTG-inducible allele of ytpQ expressed from the** ***amyE*** **locus (Strain ORB8011).** Growth curves are shown in which cultures of JH642 cells and ORB8011 cells are grown in TSS medium containing Trp and Phe auxotrophic requirements.
(TIF)

Figure S4 **Assay of** ***ytpQ*** **- and** ***ytoQ*** **-directed β-galactosidase activity in wild type cells and cells bearing the IPTG inducible** ***spx^DD^*** **allele.** A. Open circles: *ytpQ*::pMUTIN Physpank-*spx^DD^* without IPTG. Closed circles: with IPTG. B. Open triangles: *ytoQ*::pMUTIN Physpank-*spx^DD^* without IPTG. Closed triangles: with IPTG. C. The expression of the *ytpQ*- and *ytoQ-lacZ* fusions was measured in *ytpQ*::pMUTIN and *ytoQ*::pMUTIN cells in the absence of the IPTG-inducible *spx^DD^* construct. Open circles: *ytpQ*::pMUTIN without IPTG. Closed circles: *ytpQ*::pMUTIN with IPTG. Open triangles: *ytoQ*::pMUTIN without IPTG. Closed triangles: *ytoQ*::pMUTIN with IPTG.
(TIF)

Figure S5 **Hierarchical clustering analysis of gene expression profiles up- and downregulated in** ***spx*** **and** ***ytpQ*** **mutants.** Gene expression data were clustered based on the induction or repression ratios in the *spx* and *ytpQ* mutants. Nodes enriched for phage-related genes of *B. subtilis* are shown.
(TIF)

Table S1 **Expression levels of spx-controlled genes in cells bearing an IPTG-inducible allele of** ***spx*** **(** ***spx^DD^*** **) encoding a protease resistant form of Spx protein.** The values are log2 of transcript level ratio between cells grown in presence and absence of IPTG [12].
(TIF)

Table S2 **Induction and repression of genes in the** ***spx*** **and** ***ytpQ*** **mutants under control conditions and in response to 2.8 mM methylglyoxal stress as revealed by transcriptome analyses.** The averages ratios and standard deviations as well as log2-fold changes for all induced and repressed genes are calculated from three transcriptome replicate experiments at control conditions and after 10 min of exposure to 2.8 mM methylglyoxal. All genes with induction ratios of at least three-fold were listed and classified according to previously described regulons according to http://dbtbs.hgc.jp and SubtiWiki database (http://subtiwiki.uni-goettingen.de/wiki/index.php/Main_Page).
(XLS)

Table S3 **Induction and repression of genes under control conditions and in response to methylglyoxal in the** ***spx*** **and** ***ytpQ*** **mutants as revealed by transcriptome analyses.** The transcriptome datasets included log2-fold expression changes of average values of three transcriptome replicates according to Table S2. All induced and repressed genes under control and methylglyoxal stress were listed and classified into regulons according to http://dbtbs.hgc.jp and SubtiWiki database (http://subtiwiki.uni-goettingen.de/wiki/index.php/Main_Page). These data were used for the hierarchical clustering analysis presented in Figures 5 and 6.
(XLS)
