# Supplementary figures and images for: Phenotype Enhancement Screen of a Regulatory spx Mutant Unveils a Role for the ytpQ Gene in the Control of Iron Homeostasis
Source: PLoS One. 2011 Sep 20;6(9):e25066. doi: 10.1371/journal.pone.0025066 (PMC3176815; doi:10.1371/journal.pone.0025066)

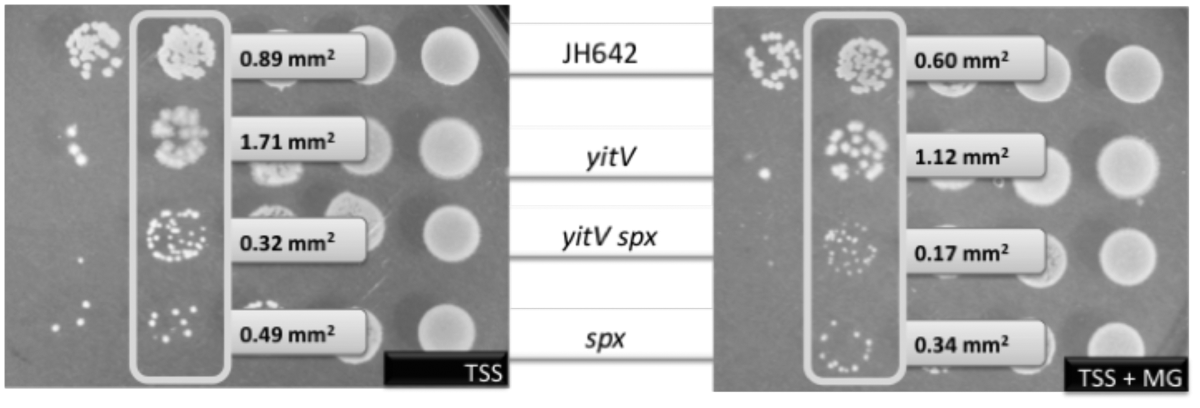

Supplement: Figure S1 — Colonies of serially diluted, spotted cultures on TSS plates with and without 3 mM methylglyoxal. Strains are JH642 (wild type parent), spx mutant, yitV, and yitV spx mutant. Values are average colony size as determined by Pixicillus. (TIF) [file pone.0025066.s001.tif]

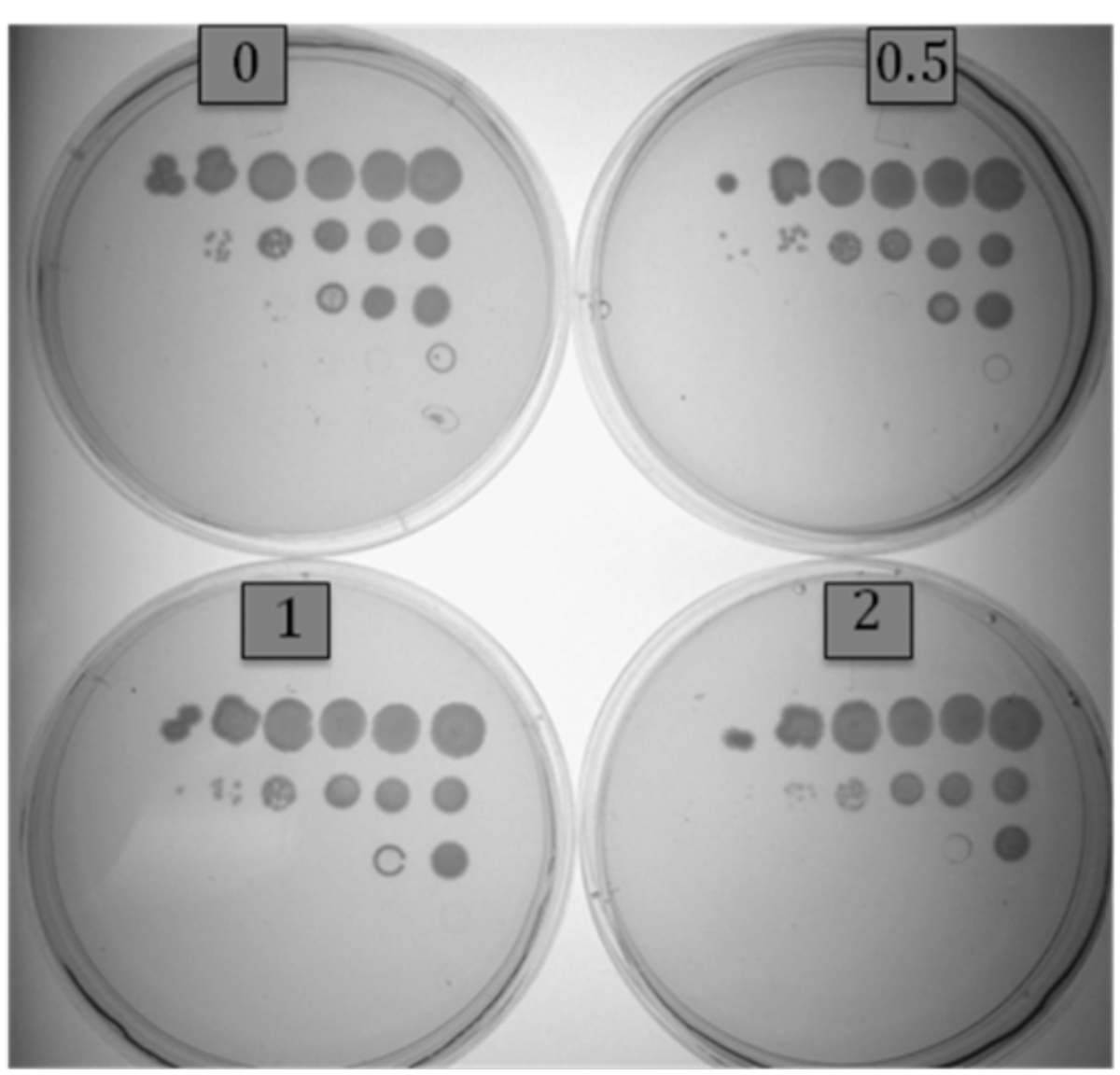

Supplement: Figure S2 — TSS minimal medium agar containing 0, 0.5, 1, and 2 mM MG. From top to bottom rows: JH642 (Wild type parent), spx::tet mutant, ytpQ deletion mutant, spx::tet ytpQ deletion double mutant. Cells were grown in TSS medium to mid-log phase, then serially diluted 10-fold. Ten µl of each dilution was then spotted onto the agar surface. Plates were incubated at 37°C. (TIF) [file pone.0025066.s002.tif]

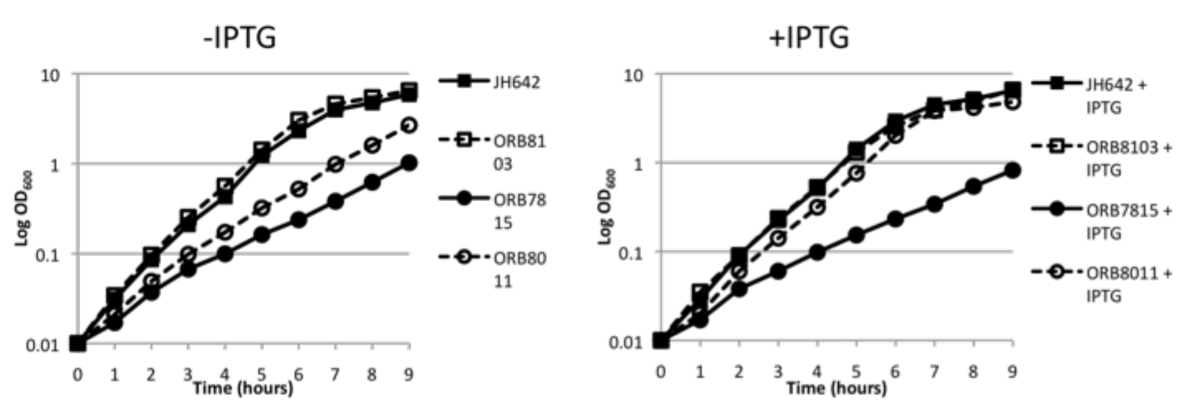

Supplement: Figure S3 — Complementation of the Δ ytpQ :: spc mutation by an IPTG-inducible allele of ytpQ expressed from the amyE locus (Strain ORB8011). Growth curves are shown in which cultures of JH642 cells and ORB8011 cells are grown in TSS medium containing Trp and Phe auxotrophic requirements. (TIF) [file pone.0025066.s003.tif]

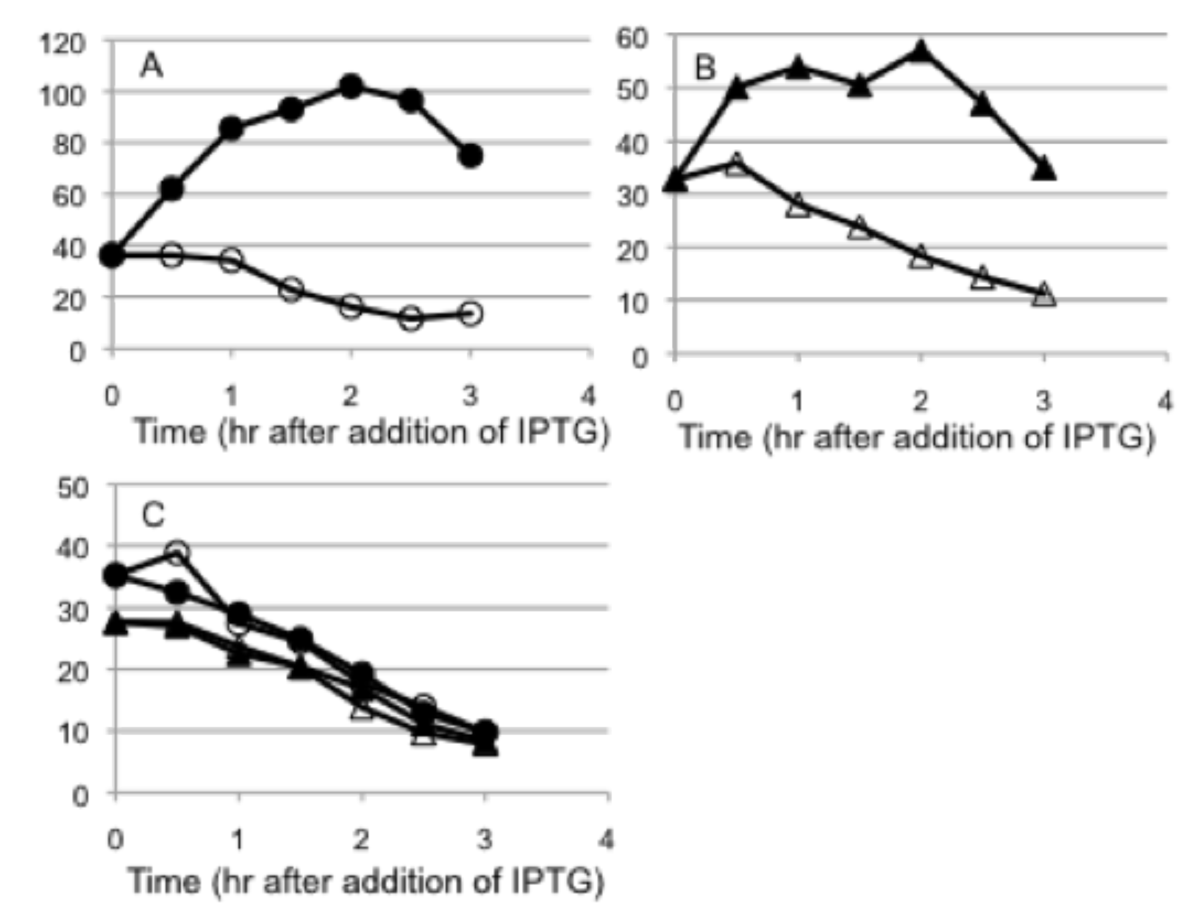

Supplement: Figure S4 — Assay of ytpQ - and ytoQ -directed β-galactosidase activity in wild type cells and cells bearing the IPTG inducible spxDD allele. A. Open circles: ytpQ::pMUTIN Physpank-spxDD without IPTG. Closed circles: with IPTG. B. Open triangles: ytoQ::pMUTIN Physpank-spxDD without IPTG. Closed triangles: with IPTG. C. The expression of the ytpQ- and ytoQ-lacZ fusions was measured in ytpQ::pMUTIN and ytoQ::pMUTIN cells in the absence of the IPTG-inducible spxDD construct. Open circles: ytpQ::pMUTIN without IPTG. Closed circles: ytpQ::pMUTIN with IPTG. Open triangles: ytoQ::pMUTIN without IPTG. Closed triangles: ytoQ::pMUTIN with IPTG. (TIF) [file pone.0025066.s004.tif]

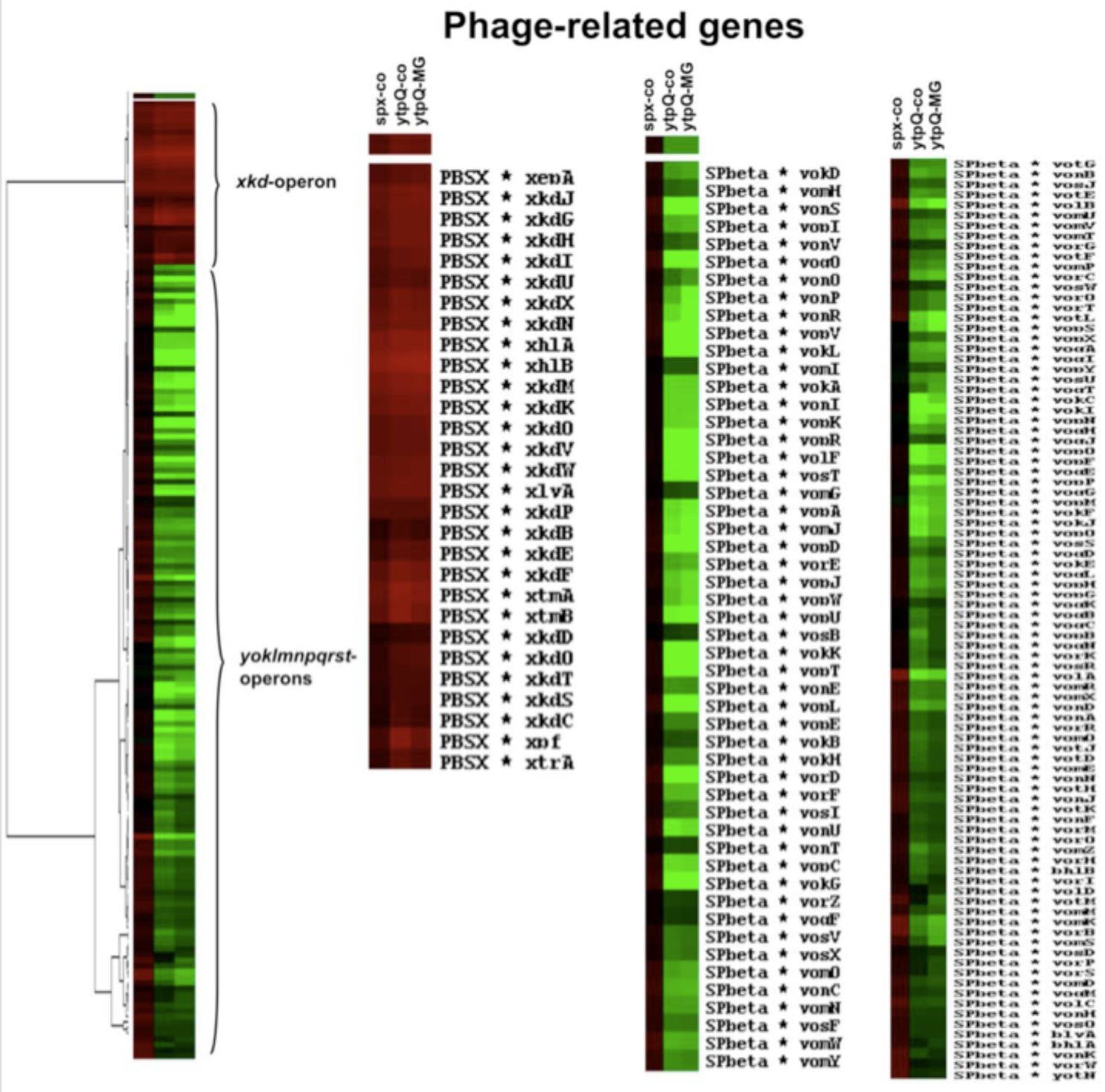

Supplement: Figure S5 — Hierarchical clustering analysis of gene expression profiles up- and downregulated in spx and ytpQ mutants. Gene expression data were clustered based on the induction or repression ratios in the spx and ytpQ mutants. Nodes enriched for phage-related genes of B. subtilis are shown. (TIF) [file pone.0025066.s005.tif]

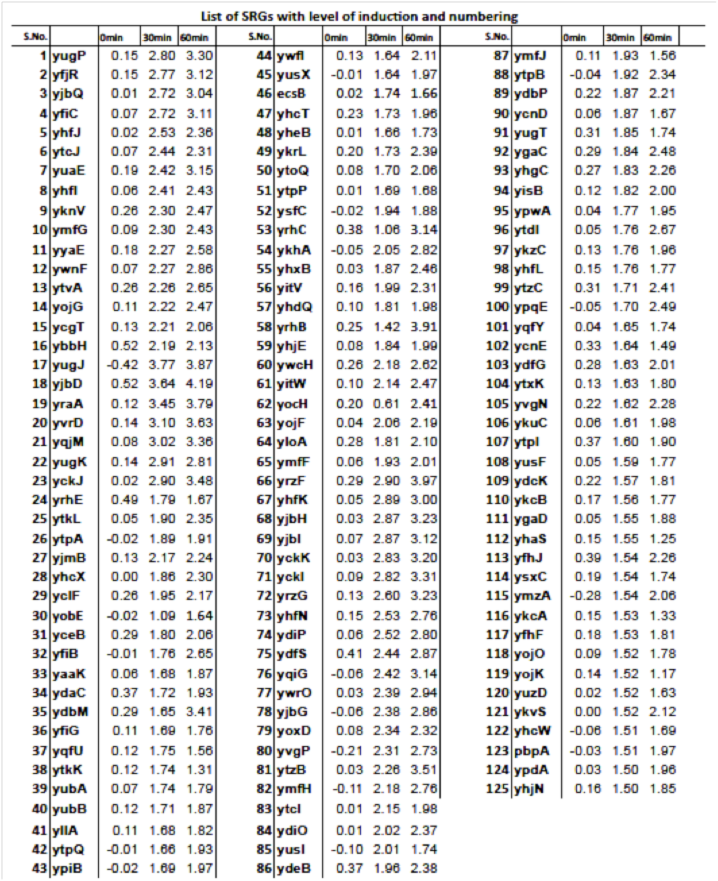

Supplement: Table S1 — Expression levels of spx-controlled genes in cells bearing an IPTG-inducible allele of spx ( spxDD ) encoding a protease resistant form of Spx protein. The values are log2 of transcript level ratio between cells grown in presence and absence of IPTG [12]. (TIF) [file pone.0025066.s006.tif]
